# Gene expression of tendon markers in mesenchymal stromal cells derived from different sources

**DOI:** 10.1186/1756-0500-7-826

**Published:** 2014-11-20

**Authors:** Janina Burk, Claudia Gittel, Sandra Heller, Bastian Pfeiffer, Felicitas Paebst, Annette B Ahrberg, Walter Brehm

**Affiliations:** Translational Centre for Regenerative Medicine (TRM), University of Leipzig, Philipp-Rosenthal-Strasse 55, 04103 Leipzig, Germany; Large Animal Clinic for Surgery, Faculty of Veterinary Medicine, University of Leipzig, An den Tierkliniken 21, 04103 Leipzig, Germany; Department of Pathology and Laboratory Medicine, Tulane University, 1430 Tulane Avenue, New Orleans, Louisiana 70112 USA; Department of Orthopedics, Traumatology and Plastic Surgery, University of Leipzig, Liebigstrasse 20, 04103 Leipzig, Germany

**Keywords:** MSC, Mesenchymal stromal cell, Tendon, Adipose tissue, Bone marrow, Umbilical cord, Collagen, Decorin, Scleraxis, Tenascin-C

## Abstract

**Background:**

Multipotent mesenchymal stromal cells (MSC) can be recovered from a variety of tissues in the body. Yet, their functional properties were shown to vary depending on tissue origin. While MSC have emerged as a favoured cell type for tendon regenerative therapies, very little is known about the influence of the MSC source on their properties relevant to tendon regeneration.

The aim of this study was to assess and compare the expression of tendon extracellular matrix proteins and tendon differentiation markers in MSC derived from different sources as well as in native tendon tissue. MSC isolated from equine bone marrow, adipose tissue, umbilical cord tissue, umbilical cord blood and tendon tissue were characterized and then subjected to mRNA analysis by real-time polymerase chain reaction.

**Results:**

MSC derived from adipose tissue displayed the highest expression of collagen 1A2, collagen 3A1 and decorin compared to MSC from all other sources and native tendon tissue (p < 0.01). Tenascin-C and scleraxis expressions were highest in MSC derived from cord blood compared to MSC derived from other sources, though both tenascin-C and scleraxis were expressed at significantly lower levels in all MSC compared to native tendon tissue (p < 0.01).

**Conclusions:**

These findings demonstrate that the MSC source impacts the cell properties relevant to tendon regeneration. Adipose derived MSC might be superior regarding their potential to positively influence tendon matrix reorganization.

**Electronic supplementary material:**

The online version of this article (doi:10.1186/1756-0500-7-826) contains supplementary material, which is available to authorized users.

## Background

Tendon disease is a common cause of acute pain and long-term mobility loss in athletes and elderly patients [1–4]. Due to the predominantly degenerative character of the disease, in many cases, regeneration following acute injury cannot be achieved by conventional therapies [1–4]. Cell-based therapies, however, were shown to have positive effects on tendon regeneration not only in small animal studies, but also in the equine large animal model [5–8].

Multipotent mesenchymal stromal cells (MSC) are currently the most frequently used cell type in tendon therapy based on their functions as connective tissue cell progenitors and potent immunomodulators [9, 10]. However, cells that correspond to the general definition of MSC can be found in many different locations in the body, and several studies showed that important differences exist between MSC derived from different sources [11–16]. Thus, it is to be expected that the MSC source also has an impact on the success of the clinical use of the cells. In order to be able to choose the optimal cell source, more knowledge is required on cell characteristics that are specifically important for the intended therapeutic application.

Aiming at regenerative tendon therapies, it is important to consider that tendons are composed of mainly extracellular matrix with a strictly hierarchical organization of collagen fibrils [4, 17]. The re-organization of these fibrils and other extracellular matrix components after injury is crucial for regaining the mechanical load capacity of the tendon and thus to prevent re-injury. MSC application was shown to improve the extracellular matrix structure of damaged tendons and increased the collagen 1 content towards normal levels [6, 7], leading to the assumption that replacement and remodelling of the extracellular matrix is a major contribution of MSC to tendon healing. Aiming to apply MSC that best possibly support the extracellular matrix remodelling, it appears of great importance to know the extent of the expression of tendon extracellular matrix components in MSC from different tissue sources.

Therefore, in this study, it was aimed to further extend and support our previous data on the comparative characterization of equine MSC from different sources [13, 18]. For this purpose, MSC derived from different tissue origins were used for a comparative assessment of their gene expression levels of molecules that are relevant to tenogenesis and to the composition and structure of tendon extracellular matrix. Cells were isolated from equine bone marrow (BM), adipose tissue (AT), tendon tissue (TT), umbilical cord blood (UCB) and umbilical cord tissue (UCT). Cells from all samples were subjected to MSC characterization assays and to real-time reverse-transcription polymerase chain reaction (RT-PCR) analyses of collagen 1A2, collagen 3A1, decorin, tenascin-C and scleraxis expression. Native tendon tissue controls (naT) were used as a reference for the gene expression analyses.

## Results

### Basic MSC characteristics

Plastic-adherent cells could be isolated from all samples. The cells were capable of adipogenic, osteogenic and chondrogenic differentiation as confirmed by Oil Red O, von Kossa or Alcian blue staining, respectively. Flow cytometry revealed that they expressed CD29 and CD44, and lacked expression of CD34, CD45 and MHCII, although there were some variations between the percentages of positive cells between donors and MSC sources (data published elsewhere [18]).

### Collagen expression

**Figure 1:**
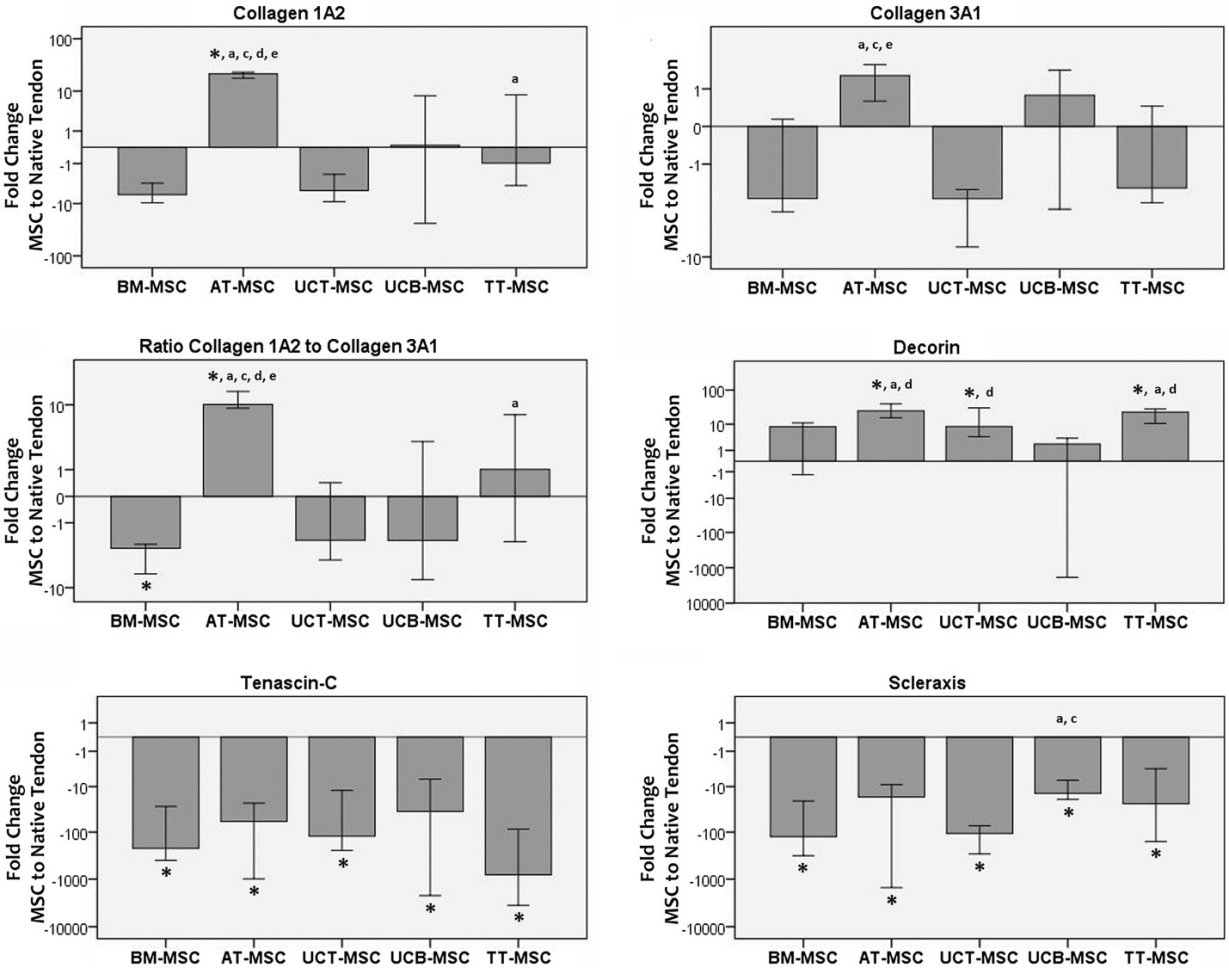
**Tendon marker expression in MSC relative to naT.** Gene expression of tendon markers in multipotent mesenchymal stromal cells (MSC) from different sources plotted as fold change to the median expression in native tendon tissue; bars indicate the median values, whiskers the 99% confidence interval; p < 0.01 compared to *) native tendon tissue control (naT); a) bone marrow (BM)-MSC; b) adipose tissue (AT)-MSC; c) umbilical cord tissue (UCT)-MSC; d) umbilical cord blood (UCB)-MSC; e) tendon tissue (TT)-MSC.

AT-MSC displayed a distinctively high collagen 1A2 expression, which differed significantly from MSC derived from all other sources or from naT (p < 0.01). BM- and UCT-MSC collagen 1A2 expression was overall lowest. Although at a lower level than collagen 1A2, AT-MSC also showed the highest collagen 3A1 expression, which was significant compared to all other sample types (p < 0.01) except for UCB-MSC. Correspondingly, the ratio of collagen 1A2 expression to collagen 3A1 expression was higher in AT-MSC than in MSC derived from all other sources or in naT (p < 0.01). BM-MSC displayed the lowest ratio (p < 0.01 compared to AT-MSC, TT-MSC and naT) (Figure 1 and Additional file 1: Figure S1, Table 1).

**Table 1:** **Tendon marker expression levels in MSC and naT**

	Target gene expression ratios (median (interquartile range))					
	Collagen 1A2	Collagen 3A1	Collagen 1A2/collagen 3A1	Decorin	Tenascin-C	Scleraxis
BM-MSC	0,0322 (0,0306)	0,4163 (0,3440)	0,1066 (0,0225)	0,8936 (0,2955)	0,0006 (0,0006)	0,0112 (0,0230)
AT-MSC	3,9673 (0,7435)*,a,c,d,e	1,6649 (0,2418)*,a,c,e	2,2667 (0,3658) *,a,c,d,e	2,4893 (1,4706)*,a,d	0,0021 (0,0033)	0,0874 (0,0896)
UCT-MSC	0,0418 (0,0364)	0,3971 (0,3039)	0,1753 (0,0630)	0,8952 (0,5497)*,d	0,0010 (0,0002)	0,0129 (0,0031)
UCB-MSC	0,1888 (1,0578)	1,1562 (0,5452)	0,1741 (0,6369)	0,2870 (0,0270)	0,0034 (0,0047)	0,1019 (0,0889) a,c
TT-MSC	0,2010 (0,3445) a	0,5891 (0,2539)	0,4111 (0,5535) a	2,2928 (1,1165)*,a,d	0,0001 (0,0001)	0,0686 (0,1441)
naT	0,1735 (0,1645)	0,6516 (0,7127)	0,2042 (0,1226) a	0,0951 (0,3832)	0,1176 (0,0369) a,b,c,d,e	16,5828 (1,6803) a,b,c,d,e

Gene expression of tendon markers normalized to ACTB and GAPDH. P < 0.01 compared to *) native tendon tissue control (naT); a) bone marrow (BM)-multipotent mesenchymal stromal cells (MSC); b) adipose tissue (AT)-MSC; c) umbilical cord tissue (UCT)-MSC; d) umbilical cord blood (UCB)-MSC; e) tendon tissue (TT)-MSC. Corresponding data are shown in the additional file (Additional file 1: Figure S1).

### Decorin expression

Decorin expression was generally higher in all MSC than in naT, with the highest expression levels in AT- and TT-MSC (p < 0.01 compared to naT and BM-MSC). Of all MSC sources, UCB-MSC displayed the lowest decorin expression (p < 0.01 compared to AT-, TT- and UCT-MSC), with one single UCB-MSC sample showing an outstandingly low expression (Figure 1 and Additional file 1: Figure S1, Table 1).

### Tenascin-C and scleraxis expression

Tenascin-C and scleraxis expression levels were both significantly higher in naT than in MSC, irrespective of their source (p < 0.01). For tenascin-C expression, no significant differences between MSC from different sources were found. Moreover, tenascin-C was expressed at overall lower levels than the other genes investigated. For scleraxis, differences were also evident between MSC from different sources, with the highest median expression in UCB-MSC (p < 0.01 compared to BM- and UCT-MSC) (Figure 1 and Additional file 1: Figure S1, Table 1).

## Discussion

In this study, we showed that significant differences exist between the gene expression of tendon extracellular matrix components in MSC derived from different sources, which is of potential relevance for MSC application in tendon therapy.

Most remarkably, there was a clear pattern that AT-MSC expressed the extracellular matrix proteins collagen 1A2, collagen 3A1 and decorin at highest levels. In contrast, collagen expression was low in BM- and UCT-MSC. Furthermore, the ratio of collagen 1A2 to collagen 3A1 was highest in AT-MSC and lowest in BM- and UCT-MSC. Collagen 1A2 is the most abundant protein in healthy tendon tissue, while collagen 3A1 is also present but higher quantities are only found during tendon repair [4, 17]. Decorin is the most abundant proteoglycan in tendon tissue and plays an important role in the regulation of the collagen fibril structure and cell proliferation as well as in stimulating immune responses [19]. MSC which highly express these important extracellular matrix proteins may therefore have the best potential to positively influence matrix reorganization during tendon healing.

Regarding the expression of tenascin-C and scleraxis, both genes being expressed at lower levels in MSC than in naT, differences between MSC samples were not as eminent as those between the expression levels of collagens or decorin. Among the MSC derived from different sources, UCB-MSC displayed the highest median expression and AT-MSC the second highest median expression of both tenascin-C and scleraxis. The glycoprotein tenascin-C is known to be present in healthy tendon and is involved in the regulation of collagen fibrillogenesis, but is also associated with tendon disease [20, 21]. However, although frequently being used as an additional tenogenic differentiation marker [22, 23], tenascin-C is expressed in a wide variety of cell types and its upregulation is also associated with non-musculoskeletal diseases such as asthma or cancer [24]. The transcription factor scleraxis is considered to be a more specific tenogenic differentiation marker, as it was found to be essential to tenogenesis, although further signals are required to contribute to tendon development [25–27]. Scleraxis was further shown to regulate collagen 1A2 synthesis in cardiac fibroblasts [28] and may therefore also play an important role in the re-organization of the extracellular tendon matrix after injury. However, in the current study using unstimulated MSC, a correlation between scleraxis expression and collagen 1A2 expression was not evident.

For healthy, adult tendon tissue, high expression levels of collagen 1A2 and scleraxis and low expression levels of tenascin-C were previously found to be most specific, while cultured tenocytes displayed a significantly lower scleraxis expression [21]. This is in accordance with the current study, in which scleraxis was expressed at significantly lower levels in all MSC compared to naT, while collagen 1A2 and collagen 3A1 expression by monolayer-cultured MSC, except for AT-MSC, did not differ significantly from naT. This supports the hypothesis that scleraxis is a useful marker to distinguish undifferentiated fibroblast-like cells and fully differentiated tenocytes.

Our findings also correspond with a previously published study in which rat BM- and TT-MSC were compared regarding their properties relevant to tendon regeneration [29]. In this study, TT-MSC displayed superior properties and a higher expression of tendon markers compared to BM-MSC [29]. While in the current study, tendon marker expression in TT-MSC was also higher than in BM-MSC, AT-MSC diplayed an even higher marker expression than TT-MSC. Therefore, based on the hypothesis that re-organization and replacement of the extracellular matrix is a major contribution of MSC to tendon healing, AT may be the most promising cell source for tendon therapy.

There are further aspects which could have an influence on therapeutic success in tendon therapy. Besides practical issues such as the required cell numbers and the optimal way of applying the cells, MSC properties such as their viability and their immunomodulatory capacity are of potential importance. Previous studies showed that differences between equine MSC from different sources also exist with regard to their viability and proliferation potential, at which AT-MSC have already been shown to be superior compared to MSC derived from other sources [13, 15]. In contrast, no major differences between equine MSC from different sources could be detected with regard to their influence on lymphocyte proliferation and cytokine expression [30], indicating a similar immunomodulatory potential.

Taking into consideration their ease of harvest, high viability and good immunomodulatory potential as well as their high expression of tendon extracellular matrix components as demonstrated in the current study, AT-MSC appear to display several advantages for clinical use in tendon therapy.

However, the current study is of preliminary character. Comparative studies of MSC from different sources using in vitro models that mimic tenogenic conditions [31] may help to further substantiate our hypothesis. Eventually, it remains to be investigated whether a high expression of extracellular matrix proteins in vitro finally leads to the intended results in terms of replacement and re-organization of the extracellular matrix after in vivo application.

## Conclusions

The current study demonstrates that MSC from different sources display different tendon marker expression patterns, particularly with respect to the most important tendon extracellular matrix components. As improvement in matrix re-organization has been shown to be the major beneficial effect of MSC in tendon therapy, this finding is of potential clinical relevance. AT-MSC showed the highest expression of tendon extracellular matrix components and may therefore be most potent to positively influence extracellular matrix re-organization after tendon injury.

## Methods

### Sample collection

For primary cell isolation, UCB and UCT were recovered from healthy foals and BM, AT and TT were recovered from healthy adult horses (age range: 3–18 years), as described previously [13] and with approval by the local ethics committee (Landesdirektion Leipzig, Germany, A13/10). Further tendon samples were collected from the superficial digital flexor tendons of animals which had been euthanized for unrelated reasons and immediately stored at -80°C to be used as the naT reference for RNA analysis.

### Cell culture and characterization

Cells were isolated by density gradient centrifugation from BM and UCB and by collagenase I digestion from AT, TT and UCT as described previously [13]. They were then seeded into culture flasks in low glucose (1 g/L) Dulbecco’s modified eagle medium (Life Technologies GmbH) supplemented with 20% fetal bovine serum (Sigma Aldrich, Taufkirchen, Germany), 1% penicillin-streptomycin (PAA Laboratories GmbH, Coelbe, Germany) and 0.1% gentamycin (Life Technologies GmbH), and incubated at 37°C, 95% humidity and 5% CO_2_ for selection of plastic-adherent cells and their further expansion. Passage 3 cells were used to confirm their trilineage differentiation potential into adipocytes, osteoblasts and chondrocytes [13], as well as for the assessment of surface marker expression by flow cytometry [18].

### RNA analysis

Five samples of UCB-MSC and 6 samples of each BM-, AT-, TT- and UCT-MSC (passage 3) as well as naT were subjected to RNA analysis by real-time RT-PCR.

Total RNA of MSC was isolated using the RNeasy® Mini Kit (Qiagen, Hamburg, Germany) according to instructions of manufacturers (protocol version 09/2010). Frozen naT samples were sliced in 12 μm sections with a microtome CM 3050 S equipped with cryochamber (Leica Microsystems, Wetzlar, Germany) and incubated for 60 min at 55°C in homogenization buffer (15 mM HEPES, 2.5 mM KCl, 68.5 mM NaCl, 450 μM Na_2_HPO_4_, 17.5 mM EDTA, 27.5 mM glucose at pH 7) containing 100 μg/ml proteinase K (Ambion® Life Technologies, Darmstadt, Germany). To homogenize the sample, lyzed tendon tissue was passed through a 20-gauge needle several times. Total RNA of tendon tissue homogenate was purified with phenol/chloroform extraction and isopropanol precipitation.

DNase-treated RNA was reverse transcribed using the RevertAid H Minus First Strand cDNA Synthesis Kit (Thermo Fisher Scientific, Dreieich, Germany) or the Omniscript RT Kit (Qiagen) with oligo-dT18 primers as described by the manufacturers. Relative quantification of cDNA was performed with a 7500 Real Time PCR System (Applied Biosystems, Foster City, USA) and SYBR® Green as double-strand DNA-specific dye (iQ™SYBR® Green Supermix, Bio-Rad Laboratories, Hercules, USA). Primers amplifying the respective genes are listed in Table 2 and corresponding Ct values were used to analyze gene expression. RT-PCR analysis of scleraxis expression was performed with two different primer sets for native tendon tissue and isolated MSC. To confirm that relative quantification of both sample types was comparable, expression of scleraxis in MSC derived from BM, AT, UCT, UCB and TT was analyzed using both primer sets. Obtained data showed that relative expression of scleraxis was similar in MSC derived from different sources irrespective of the primer set used (data not shown). Therefore, scleraxis mRNA expression levels of all samples can be compared.

**Table 2:** **Primer sequences**

Equine gene	Forward primer	Reverse primer	Accession number	PCR product in bp
ACTB	ATCCACGAAACTACCTTCAAC	CGCAATGATCTTGATCTTCATC	NM_001081838.1	174
GAPDH	TGGAGAAAGCTGCCAAATACG	GGCCTTTCTCCTTCTCTTGC	NM_001163856.1	309
Collagen 1A2	CAACCGGAGATAGAGGACCA	CAGGTCCTTGGAAACCTTGA	XM_001492939.1	243
Collagen 3A1	AGGGGACCTGGTTACTGCTT	TCTCTGGGTTGGGACAGTCT	XM_001917620.2	216
Decorin	ACCCACTGAAGAGCTCAGGA	GCCATTGTCAACAGCAGAGA	NM_001081925.2	239
Tenascin-C	TCACATCCAGGTGCTTATTCC	CTAGAGTGTCTCACTATCAGG	XM_001916622.2	163
Sleraxis_naT_	TACCTGGGTTTTCTTCTGGTCACT	TATCAAAGACACAAGATGCCAGC	NM_001105150.1	51
Scleraxis_MSC_	AGAGAAAGTTGAGCAAGGACC	TCAAAGACACAAGATGCCAGC	NM_001105150.1	294

Primer sequences used for real-time reverse transcription polymerase chain reaction.

Primer efficiency (E_gene_) was calculated after serial dilution of template cDNA and target gene expression levels were normalized to the reference genes ACTB and GAPDH as described previously [32]:


Further, fold changes (FC) of MSC gene expression compared to naT gene expression were calculated from the normalized gene expression ratios:


### Statistical analysis

Kruskall-Wallis one way analyses of variance and subsequent Mann–Whitney-U tests were performed to analyze differences between the normalized gene expression levels of the sample groups. P < 0.01 was considered as significant.

## Electronic supplementary material

Additional file 1: Figure S1: Tendon marker expression levels in MSC and naT. Gene expression of tendon markers in multipotent mesenchymal stromal cells (MSC) from different sources and in native tendon tissue (naT), given as ratios normalized to ACTB and GAPDH. BM: bone marrow; AT: adipose tissue; UCB: umbilical cord blood; UCT: umbilical cord tissue; TT: tendon tissue. (TIFF 960 KB)

## Abbreviations

MSC: Multipotent mesenchymal stromal cell(s)
BM: Bone marrow
AT: Adipose tissue
TT: Tendon tissue
UCB: Umbilical cord blood
UCT: Umbilical cord tissue
naT: Native tendon tissue control
RT-PCR: Reverse-transcription polymerase chain reaction
FC: Fold change(s).
