# Supplementary figures and images for: Gene expression of tendon markers in mesenchymal stromal cells derived from different sources
Source: BMC Res Notes. 2014 Nov 20;7:826. doi: 10.1186/1756-0500-7-826 (PMC4247609; doi:10.1186/1756-0500-7-826)

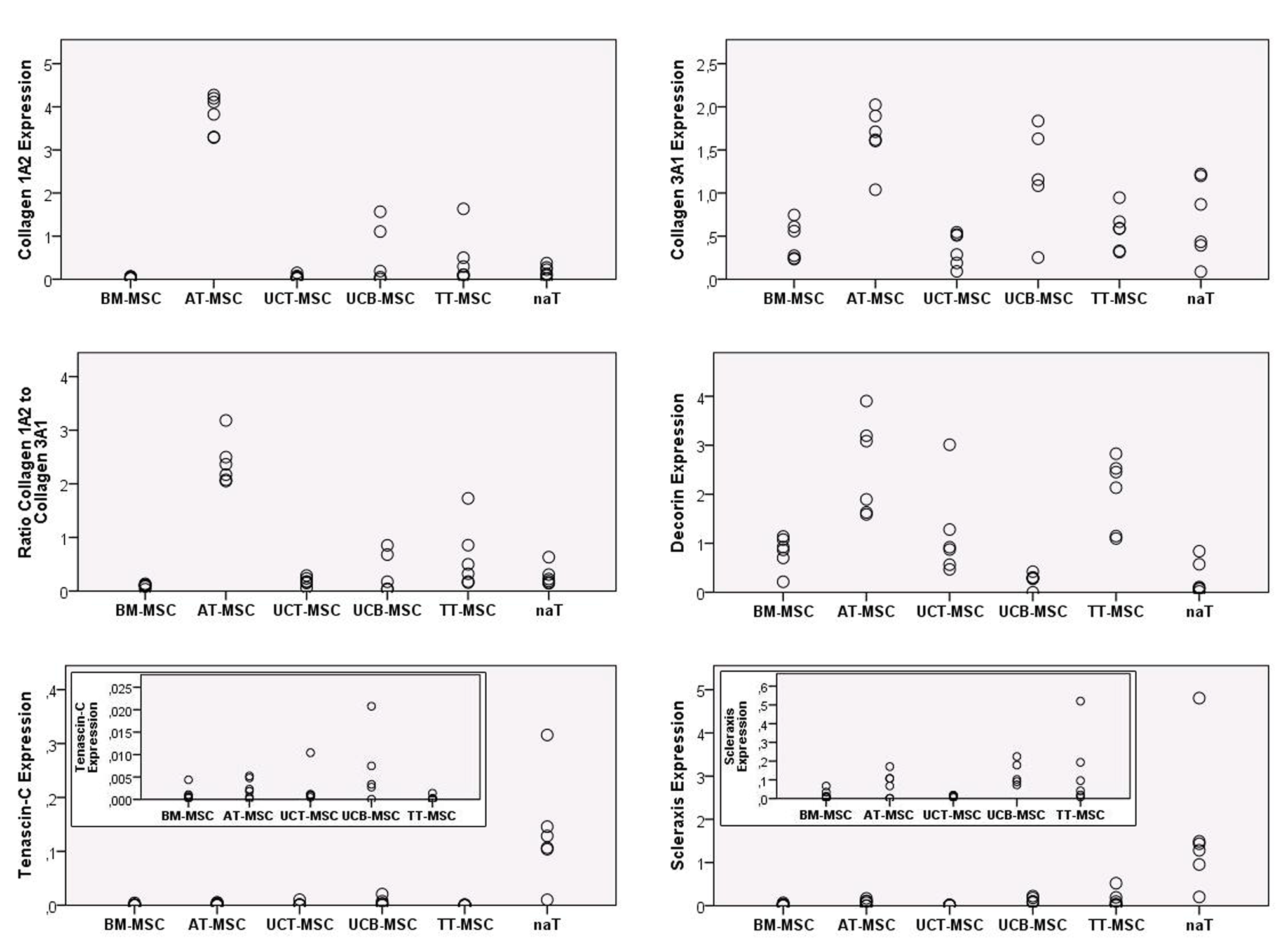

Supplement: Supplementary file 1 — Additional file 1: Figure S1: Tendon marker expression levels in MSC and naT. Gene expression of tendon markers in multipotent mesenchymal stromal cells (MSC) from different sources and in native tendon tissue (naT), given as ratios normalized to ACTB and GAPDH. BM: bone marrow; AT: adipose tissue; UCB: umbilical cord blood; UCT: umbilical cord tissue; TT: tendon tissue. (TIFF 960 KB) [file 13104_2014_3346_MOESM1_ESM.tiff]
